# PICALM::MLLT10 translocated leukemia

**DOI:** 10.1002/1873-3468.70279

**Published:** 2026-01-14

**Authors:** John M. Cullen, Antonia C. Nakatsugawa, Natalie Barton, Henry Haines, Gary S. Stein, Janet L. Stein, Daniel S. Wechsler, Jessica L. Heath

**Affiliations:** ^1^ Department of Pediatrics University of Vermont Larner College of Medicine Burlington USA; ^2^ Department of Biochemistry University of Vermont Larner College of Medicine Burlington USA; ^3^ University of Vermont Cancer Center Burlington USA; ^4^ Aflac Cancer & Blood Disorders Center, Children's Healthcare of Atlanta USA; ^5^ Department of Pediatrics Emory University School of Medicine Atlanta USA

**Keywords:** CALM‐AF10, epigenetic control, leukemia, leukemia translocation, *PICALM::MLLT10*

## Abstract

The t(10;11)(p13;q14‐21) *PICALM::MLLT10* chromosomal translocation results in the production of the CALM‐AF10 fusion oncoprotein and is a driver mutation in both acute myeloid and T‐lymphoblastic leukemia. *PICALM::MLLT10* translocated leukemia is primarily an epigenetically driven disease. Global hypomethylation results in genomic instability, while focal H3K79 hypermethylation at target genes induces cell proliferation and blocks differentiation. Nucleocytoplasmic shuttling of CALM‐AF10 and its protein partners and impaired endocytosis at the plasma membrane further influence the leukemic phenotype. Leukemias characterized by *PICALM::MLLT10* have historically been recognized to portend a poor prognosis; however, insights from larger patient cohorts provide refinement to the prognostic relevance of this chromosomal translocation, highlighting chemotherapy resistance in this leukemic subtype. In addition, a deeper biological understanding of the disease hints at potential therapeutic targets. This approach is demonstrated in the recent promising results achieved utilizing venetoclax, a BCL2 inhibitor, in patients with *PICALM::MLLT10* acute leukemia. Herein, we provide updates on the pathophysiology, clinical presentation, prognosis, and treatment of *PICALM::MLLT10* acute leukemia.

## Graphical abstract

This comprehensive review of *PICALM::MLLT10* translocated acute leukemia provides an in‐depth review of the structure and function of CALM, AF10, and the fusion oncoprotein (1). The multifaceted molecular mechanisms of oncogenesis, including nucleocytoplasmic shuttling (2), epigenetic modifications (3), and disruption of endocytosis (4), are then covered in detail. Finally, up‐to‐date clinical implications and treatment options are presented (5). Created in BioRender. Heath, J. (2025) https://BioRender.com/m1q6uqc.

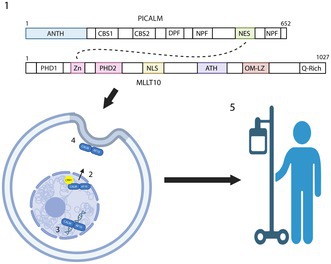

## Abbreviations

ALAL, acute leukemia of ambiguous lineage

ALL, acute lymphoblastic leukemia

AML, acute myeloid leukemia

CNS, central nervous system

DNA, deoxyribonucleic acid

EFS, event free survival

EGFR, epidermal growth factor receptor

ETP, early T‐precursor

Hyper‐CVAD, hyperfractionated cyclophosphamide, vincristine, doxorubicin, and dexamethasone

MEF, murine embryonic fibroblast

MPAL, mixed phenotype acute leukemia

NES, nuclear export sequence

NLS, nuclear localization sequence

OS, overall survival

PHD, plant homeodomain

TCR, T‐cell receptor

Tf, transferrin

TfR, transferrin receptor

VP, vincristine prednisone

WBC, white blood cell

The t(10;11)(p13;q14‐21) chromosomal translocation fuses *PICALM* (encoding the phosphatidylinositol‐binding clathrin assembly lymphoid myeloid protein (PICALM or CALM)) to *MLLT10* (encoding the AF10 protein), with the *PICALM::MLLT10* gene fusion resulting in the production of the leukemogenic CALM‐AF10 fusion protein. The past 40 years have led to increased understanding of the mechanisms underlying leukemogenesis driven by the *PICALM::MLLT10* translocation. This review will first provide a comprehensive overview of the structural domains of CALM‐AF10, relating those domains to its known functions. We then examine mechanisms of CALM‐AF10‐mediated leukemogenesis; first exploring how subcellular localization and nucleocytoplasmic shuttling might contribute to known mechanisms of CALM‐AF10 leukemogenesis, then examining the critical role that epigenetic alterations play in driving cancer development in this leukemic subtype. Finally, we review the clinicopathologic features of *PICALM::MLLT10* translocated leukemia and discuss the most up‐to‐date findings regarding prognosis and treatment options. *PICALM::MLLT10* translocated leukemias are associated with poor prognosis and poor response to induction chemotherapy. Identification of new therapeutic modalities is necessary to improve survival and decrease the burden of morbidity and mortality in *PICALM::MLLT10* translocated leukemia, and a deep understanding of the biology of the disease is the path toward the discovery of novel, rationally targeted therapies.

## Structure and function

Specific structural domains of each individual component of the CALM‐AF10 fusion oncoprotein contribute to its cellular functions. The structure and function of full‐length CALM and AF10 will be discussed first, giving context to the critical role that the functional domains of CALM and AF10 play in the known mechanisms of *PICALM::MLLT10*‐mediated leukemogenesis.

### Structure and function of CALM

The *PICALM* gene, also known as *CALM* (clathrin assembly lymphoid myeloid), encodes the CALM protein [1]. *CALM* is located on chromosome 11, contains 23 exons, and is subject to extensive alternative splicing [2, 3]. This alternative splicing can produce as many as 15 isoforms of endogenous CALM, two of which are ubiquitously expressed in humans [2]. The longest of these two isoforms contains 652 amino acids and has a molecular weight of approximately 72 kDa, while the shorter isoform of CALM lacks 49 amino acids and has a molecular weight closer to 66 kDa [4]. CALM plays an important role in facilitating clathrin‐mediated endocytosis (CME), including receptor‐mediated endocytosis. CALM promotes CME by acting as a scaffold at endocytic vesicles, binding to both clathrin and adaptor protein 2 (AP2) at the plasma membrane [5, 6]. Clathrin and AP2 drive vesicle formation and link this formation to specific substrates; the binding of CALM to both clathrin and AP2 is thought to promote vesicle formation [5, 7]. Mutations in CALM impair the formation of endocytic vesicles, resulting in diminished endocytosis of both transferrin receptors (TfRs) and epidermal growth factor receptors (EGFRs) [8, 9]. The inefficient endocytosis of TfRs in *PICALM::MLLT10* transformed cell lines results in iron deficiency *in vitro* and impairs cell proliferation upon the addition of iron chelators [8, 10]. While CALM can facilitate CME, it is not essential for CME in all cell types. CALM appears to have a disproportionate effect on CME in both synaptic vesicles of the brain, where it is associated with Alzheimer's disease, as well as in erythrocytes [2, 11].

**Fig. 1:**
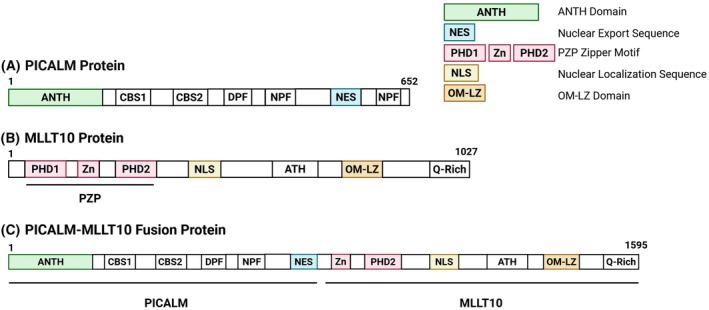
Structural domains of (A) CALM (PICALM), (B) AF10 (encoded by *MLLT10*), and (C) the CALM‐AF10 fusion oncoprotein. Important structural domains of all three proteins are highlighted in color. The domains critical for leukemogenesis include the ANTH domain and nuclear export sequence (NES) of CALM, and the PZP zipper, nuclear localization sequence (NLS), and OMLZ domain of AF10. All of these domains are retained in the CALM‐AF10 fusion. Created in BioRender. Heath, J. (2025) https://BioRender.com/c9huzi1

CALM contains multiple functional domains that promote its ability to facilitate CME (Fig. 1A) [1]. At its N terminus, CALM contains an AP180 N‐terminal homology (ANTH) domain, which mediates binding of CALM to phosphatidylinositol (PI) at the plasma membrane and vesicular soluble N‐ethylmaleimide‐sensitive factor activating protein receptor (V‐SNARE) proteins at sites of endocytosis [5, 12]. Additionally, CALM contains two clathrin binding sites (CBSs) downstream of its ANTH domain, known as CBS1 and CBS2, which are required for the binding of CALM to clathrin [1]. CALM also contains an aspartate‐proline‐phenylalanine (DPF) motif, which is thought to facilitate its binding to AP2 during endocytic vesicle formation. Adjacent to CALM's DPF motif are two asparagine‐proline‐phenylalanine (NPF) motifs, which allow CALM to recognize proteins containing Eps15 homology (EH) domains. EH domains are highly conserved among several proteins associated with CME; however, it is not well understood how CALM interacts with EH domain‐containing proteins to promote endocytosis [2, 13]. A nuclear export signal (NES) is also located between amino acid residues 520–583 on the C terminus of CALM [5, 14]. This NES functions to keep CALM primarily localized in the cytoplasm; however, CALM does translocate to the nucleus [14]. While the function of CALM in the nucleus is currently unknown, its nuclear export has been shown to be facilitated by the chromosomal maintenance 1 (CRM1) nuclear export protein [15, 16].

### Structure and function of AF10

*MLLT10* (mixed‐lineage leukemia translocated to 10) encodes a cofactor of the histone 3 lysine 79 (H3K79) methyltransferase DOT1L (disruptor of telomeric silencing‐1). *MLLT10* has 24 exons that encode the AF10 protein, which contains 1027 amino acid residues and has a molecular weight of 109 kDa [1, 17]. AF10 is one of several cofactors of the DOT1L complex, which includes AF9, AF17, and ENL [18]. While other proteins have been identified as interacting with the DOT1L complex, including Skp1, TRRAP, and β‐catenin, it is unknown whether these latter proteins are true members of the complex or rather part of the DOT1L interactome [19]. DOT1L is the only H3K79 methyltransferase known to exist in humans, and it catalyzes the mono‐, di‐, and trimethylation of H3K79 [20]. Histone modifications of H3K79 are unique in that the lysine residue is not located on an amino tail of H3 but rather is found on the surface of the histone core [20]. Di‐ and trimethylation of H3K79 are associated with active gene transcription, and DOT1L has been shown to promote both transcriptional initiation and elongation via the recruitment of RNA polymerase II to target gene promoters [18]. Knockdown of *MLLT10* reduces H3K79 di‐ and trimethylation, suggesting that AF10 may regulate or facilitate the methyltransferase activity of DOT1L [21, 22].

AF10 contains numerous domains that mediate its interactions within the nucleus (Fig. 1B). The PZP domain at the N terminus of AF10 contains two plant homeodomains (PHDs) located between residues 22–74 and 112–197, and a zinc‐knuckle in the middle of these PHDs at residues 75–111 [23, 24]. The entire PZP domain of AF10 has been shown to bind to residues 15–34 of H3, with both methylation and acetylation of H3K27 inhibiting this interaction [23]. Recognition of unmodified H3K27 by AF10's PZP domain is thought to help target DOT1L to chromatin and localize it closer to its H3K79 substrate, which is more difficult to access due to its presence on the histone core rather than at the histone tail [22]. AF10 also contains a nuclear localization signal downstream of its PZP domain, as well as an AT‐hook motif, which mediates binding to cruciform DNA [25, 26]. Near its C terminus between amino acid residues 677–758, AF10 has an octapeptide motif/leucine zipper (OM/LZ) domain. The OM/LZ domain is responsible for AF10's interactions with multiple proteins, including DOT1L, the chromatin remodeling protein GAS41, and the B/T‐cell regulator IKAROS [1, 26, 27, 28]. The end of the C terminus of AF10 contains a glutamine‐rich (Q‐rich) region [1].

### Structure of the CALM‐AF10 oncoprotein

The *PICALM::MLLT10* fusion gene encodes the CALM‐AF10 oncoprotein, which has 1595 amino acids and a molecular weight of approximately 170 kDa [29]. The CALM‐AF10 fusion protein joins all but the last four amino acid residues of CALM to residues 81–1027 of AF10 (Fig. 1C) [30]. This truncated version of AF10 in the CALM‐AF10 fusion protein results in the loss of PHD1 from the PZP domain of AF10 [23]. While the first PHD is lost in the fusion protein, CALM‐AF10 retains the second PHD, NLS, OM/LZ domain, and Q‐rich region of endogenous AF10 [1]. CALM‐AF10 maintains all the functional domains of endogenous CALM except for its second NPF motif. The OM/LZ domain of AF10 has been shown to be both necessary and sufficient for *PICALM::MLLT10*‐mediated leukemogenesis, along with the clathrin binding domain of CALM, which spans residues 400–652 [26]. CALM‐AF10 mutants in which only the clathrin binding domain of CALM is fused to the OM/LZ domain of AF10 demonstrate increased nuclear localization of CALM‐AF10 and enhanced leukemogenic transformation [26]. CALM‐AF10 typically localizes to the cytoplasm; however, both endogenous CALM and CALM‐AF10 are known to interact with the CALM‐interacting protein expressed in thymus and spleen (CATS) within the nucleus [31]. While the function of CATS is undescribed, it has been shown to be highly expressed in actively proliferating cells [31]. CALM‐AF10 also maintains CALM's nuclear export sequence (NES), located within the clathrin‐binding domain of CALM. CALM‐AF10 mutants lacking an NES show decreased leukemogenic potential and increased nuclear localization, demonstrating that the interaction between CALM‐AF10 and CRM1 is required for leukemogenesis [14]. Mutant cells expressing CRM1‐AF10 fusion proteins also show similar transcriptional phenotypes to CALM‐AF10 mutants, suggesting that CRM1 may influence AF10's localization within the nucleus [14, 16, 32].

## CALM‐AF10, CRM1 and the role of nucleocytoplasmic shuttling

Subcellular and subnuclear protein localization play important roles in modulating the function of proteins. The subcellular localization of the CALM‐AF10 fusion protein is dependent, in part, on the normal localizations of the individual CALM and AF10 proteins. While the CALM protein is known to be shuttled from the nucleus to the cytoplasm and plays important roles at the plasma membrane, AF10 contains nuclear localization sequences [15], and resides primarily within the nucleus.

### CALM‐AF10 in the cytoplasm

**Fig. 2:**
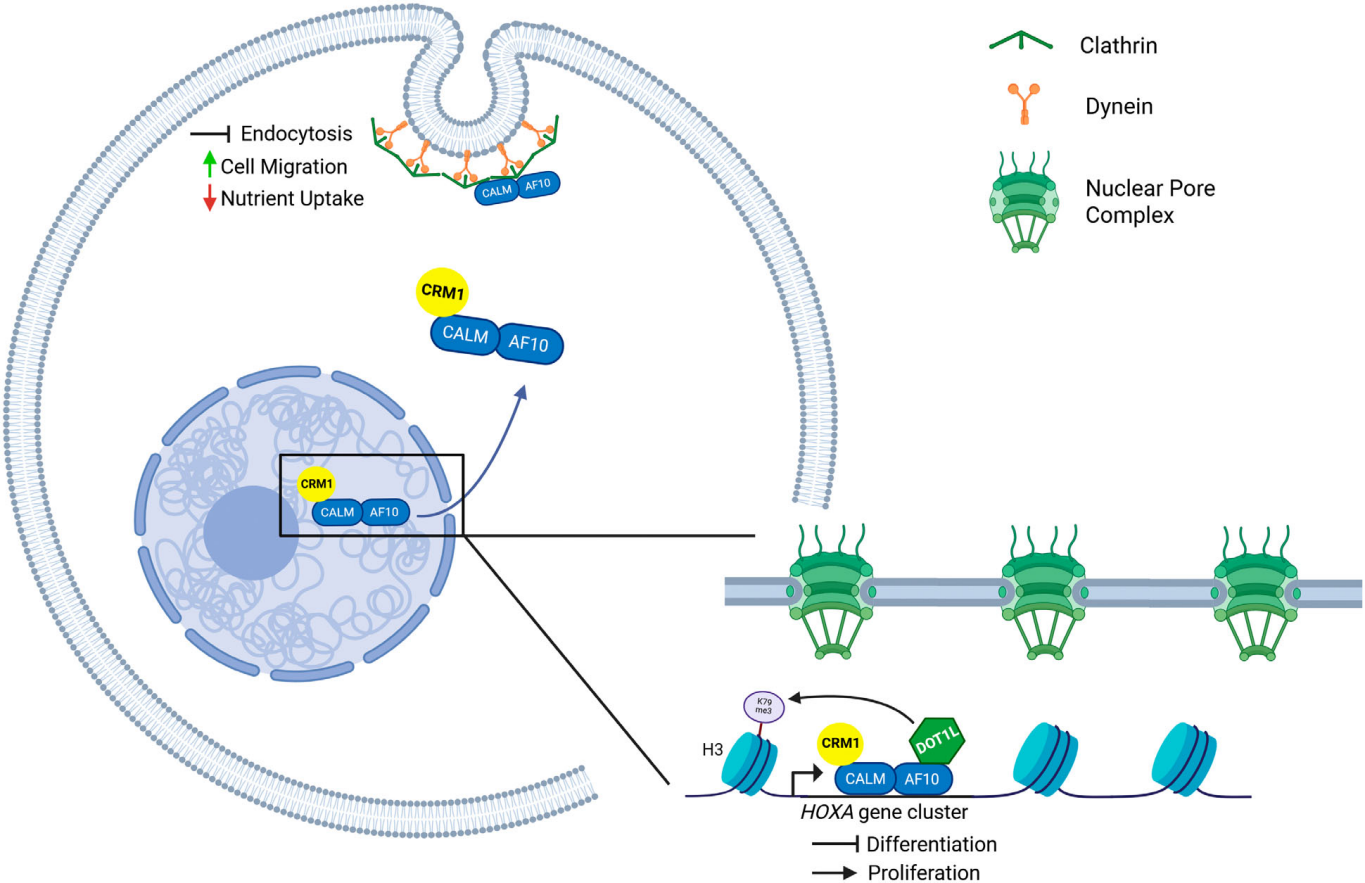
Mechanisms of CALM‐AF10‐mediated leukemogenesis. CALM‐AF10 interacts with CRM1 and DOT1L in the nucleus, resulting in focal hypermethylation of H3K79 and upregulation of critical genes, including those at the *HOXA* cluster locus, which enhance cell proliferation and block differentiation. CALM‐AF10 is transported out of the nuclear pore via its interaction with CRM1. CALM‐AF10 also interacts with clathrin and dynein at the plasma membrane, altering the dynamics of endocytic vesicle formation and clathrin‐mediated endocytosis. The role of CALM‐AF10 at the plasma membrane is still being elucidated but is known to impact nutrient uptake and cell migration. Created in BioRender. Heath, J. (2025) https://BioRender.com/mlnsmgy

CALM‐AF10 has been found at the plasma membrane, within the cytoplasm and in the nucleus, and in fact shuttles between the nucleus and the cytoplasm (Fig. 2). Archangelo *et al*. [31] fluorescently tagged CALM and CALM‐AF10 proteins and identified YFP‐CALM primarily in the cytoplasm and at the plasma membrane. Similarly, YFP‐CALM‐AF10 was overwhelmingly found in the cytoplasm, although some protein was observed in the nucleus as well [31]. Conway *et al*. [14] additionally demonstrated that CALM‐AF10 localizes predominantly to the cytoplasm via immunofluorescence experiments using HEK293 cells and murine *PICALM::MLLT10+* leukemic cells. Furthermore, Okada *et al*. [33] investigated hDOT1L and its interaction with CALM‐AF10: immunofluorescence revealed a primarily cytoplasmic localization of CALM‐AF10 in U2OS cells with some nuclear distribution. Suzuki *et al*. [34] also investigated the localization of CALM‐AF10 in COS‐7 cells and found that both CALM and CALM‐AF10 localize in the cytoplasm.

Stoddart *et al*. studied the localization of two CALM‐AF10 variants created by breakpoints in CALM representing two of the eight alternative *PICALM::MLLT10* fusion transcripts that have been identified. In CALM‐2091‐AF10, the AF10 portion replaces the last four amino acids of CALM while CALM‐1926‐AF10 is missing the 165 C‐terminal amino acids of CALM. These two CALM‐AF10 variants were analyzed utilizing GFP‐tagged constructs in 293 T cells, revealing that CALM‐1926‐AF10‐GFP localized diffusely within the cytoplasm while CALM‐2091‐AF10‐GFP clustered sporadically and at distinct sites within the cytoplasm [35].

Greif *et al*. described the association of CALM‐AF10 with IKAROS by utilizing GST‐pull down assays and co‐immunoprecipitation. The localization of IKAROS in these cells was centered around dense centromeric heterochromatin in the nucleus when expressed alone, but became both nuclear and cytoplasmic when CALM‐AF10 was present. IKAROS and CALM‐AF10 also exhibited colocalization in the cytoplasm under these conditions. Although indirect, these findings again demonstrate that CALM‐AF10 localizes to the cytoplasm [17].

### Nuclear translocation and the role of CRM1

When exposed to the nuclear export inhibitor leptomycin B (LMB), CALM and CALM‐AF10 accumulate within the nucleus [14, 35, 36]. As reported by Conway *et al*., [14] CALM‐AF10 becomes entirely nuclear in the presence of LMB in HEK293 cells and in a murine *PICALM::MLLT10+* leukemia cell line. Stoddart *et al*. [35] demonstrated that the variants CALM‐1926‐AF10‐GFP and CALM‐2091‐AF10‐GFP also translocated to the nucleus after addition of LMB; CALM‐1926‐AF10‐GFP demonstrated a diffuse nuclear distribution, while CALM‐2091‐AF10‐GFP showed a punctate pattern. The ability of LMB to alter the location in which CALM‐AF10 predominantly accumulates further implies that these proteins move between the nucleus and the cytoplasm, and specifically implicates chromosomal regional maintenance protein (CRM1) in the shuttling of CALM and CALM‐AF10 from within the nucleus to the cytoplasm. CRM1 is a nuclear export receptor, which mediates the export of leucine‐rich NES‐containing proteins through the nuclear pore complex into the cytoplasm [37]. Conway *et al*. identified a NES within the CALM portion of CALM‐AF10 between amino acids 544–553 that is recognized by CRM1. Point mutated versions of the NES within CALM‐AF10 resulted in localization solely within the nuclear compartment of HEK 293 cells. Fusing CALM's NES exclusively in‐frame with AF10 resulted in cytoplasmic localization, and treating cells expressing this version of the protein with LMB resulted in a nuclear localization pattern [14]. Additionally, CALM‐AF10 mutants with their NES removed localized to the nucleus in COS‐7 cells [34].

CRM1 interacts with the nuclear pore complex, which serves as the gateway between the nucleus and the cytoplasm. It contains nucleoporins (NUPs), a protein family that interacts with CRM1 to transport NES‐containing cargo out of the nucleus. Some NUPs serve as a scaffold for the nuclear pore complex while others regulate which molecules pass through the complex. Nucleoporins that contain phenylalanine‐glycine repeats (FG‐NUPs) are known to interact with CRM1 and act as a docking site and regulator of the nuclear pore [38].

A CRM1‐AF10 chimeric protein that replaces CALM with CRM1 mimics *PICALM::MLLT10* translocated leukemia and suggests that CALM may recruit CRM1 to critical chromatin sites. Aumann *et al*. [32] determined that mutating residues of CRM1 that interact with motifs of the NUP214 nucleoporin decreased the affinity of CRM1 for NUP214, though CRM1 remained functional. Mutating these motifs in a CRM1‐AF10 fusion protein resulted in transduced murine fetal liver hematopoietic cells, which were not able to self‐renew or form secondary or tertiary colonies in a methylcellulose colony‐forming assay. Transplanting these same cells into irradiated mice failed to result in the development of leukemia over a 300‐day period, compared with unmutated *CRM1‐AF10* transduced cells, which formed primary leukemias in 45–150 days. Further investigation revealed that impaired interaction between NUP214 and the mutated CRM1‐AF10 fusion protein may be a mechanism for the inability of the mutated fusion protein to effect transformation [32]. A mutated NUP214, in which FG motifs were mutated preventing proper interaction with CRM1, resulted in impaired binding of mutated NUP214 to *HOXA* and *MEIS1* genes, an event known to be critical for leukemogenesis [38]. Oka *et al*. [39] studying the role of Nup98 fusion proteins in leukemogenesis, showed that the CRM1 protein can specifically recruit Nup98‐HoxA9 fusion proteins to the *Hox* cluster genes, causing aberrant *Hox* gene regulation. These studies suggest a potential role for NUPs in potentiating leukemogenesis through the CRM1 nuclear export receptor in *PICALM::MLLT10* leukemias.

CRM1 is known to transport nuclear factors to the cytoplasm, thus altering gene regulation; however, recent investigations have found that CRM1 can bind *HOXA* chromatin in a pattern that resembles CALM‐AF10 binding to *HOXA* chromatin in MEFs and human leukemia cells [15]. In the absence of CALM‐AF10, CRM1 was also observed to bind *HOXA* chromatin, suggesting that CALM‐AF10 could potentially be recruited by CRM1 to the *HOXA* gene locus, though this remains an area for future detailed investigation [15]. *MEIS1*, another CALM‐AF10 target gene, was also observed to be a direct binding partner for CRM1 [32].

The CALM NES has been identified to be both necessary and sufficient for leukemogenic transformation. Transduction of the CALM NES fused to AF10 into hematopoietic precursor cells (HPCs) results in myeloid immortalization as reflected in the ability of these cells to produce secondary and tertiary colonies in colony formation assays [14]. The shuttling of CALM‐AF10 between the nucleus and cytoplasm clearly plays an important role in leukemogenesis, and the role of CRM1 in the transcriptional activation of CALM‐AF10 target genes and leukemia development is still being explored.

## Epigenetic alterations in CALM‐AF10 leukemia

*PICALM::MLLT10* translocated leukemia is an epigenetically driven disease. The hallmark epigenetic feature of this leukemia is global hypomethylation of H3K79 and local hypermethylation of H3K79 at *HOXA* genes [30]. The global loss of H3K79 methylation promotes genome instability and a propensity toward the acquisition of additional mutations [40], while the focal H3K79 hypermethylation at CALM‐AF10 target genes promotes cell proliferation and inhibits differentiation [33, 41].

### Global H3K79 hypomethylation

The histone methyltransferase Dot1‐like (DOT1L) catalyzes the mono‐, di‐, and trimethylation of lysine (K) 79 on the globular domain of histone H3 (H3K79), epigenetic marks of actively transcribed genes [42, 43, 44, 45]. It is the mammalian homolog of Dot1, the H3K79 methyltransferase originally identified in yeast, and belongs to a distinct class of histone methyltransferases characterized by the absence of a SET domain [43, 46]. In addition to transcription, DNA repair, cell cycle regulation, and development, DOT1L is vital for embryonic and adult hematopoiesis and has been implicated in leukemogenesis [47, 48, 49, 50, 51, 52, 53]. For example, *KMT2A*‐rearranged leukemias exhibit aberrant H3K79 methylation at KMT2A::MLLT3 target genes [54, 55]. DOT1L is a known cofactor of AF10 and is therefore potentially involved in *PICALM::MLLT10* leukemogenesis [56]. Recent studies suggest that the interaction between CALM‐AF10 and DOT1L contributes to leukemogenesis by altering H3K79 methylation patterns to induce genomic and transcriptional changes.

Global H3K79 hypomethylation, a feature of *PICALM::MLLT10* leukemia, is caused by CALM‐AF10‐mediated DOT1L mislocalization [40]. Lin *et al*. demonstrated that CALM‐AF10 drives H3K79 hypomethylation by competing with AF10 for DOT1L in a dominant‐negative fashion, disrupting AF10‐mediated recruitment of DOT1L to chromatin [40]. The DOT1L‐binding OM‐LZ domain on AF10 is necessary for CALM‐AF10‐mediated H3K79 hypomethylation, suggesting that CALM‐AF10 directly interacts with DOT1L to produce this epigenetic alteration [27, 40, 57]. Expanding these findings, Conway *et al*. showed that approximately half of mouse embryonic fibroblasts (MEFs) stably transduced with *PICALM::MLLT10* displayed H3K79 hypomethylation, as well as colocalization of CALM‐AF10 and DOT1L in the cytoplasm [14]. It is likely that the CALM NES, located between amino acids 544–553, is a major driver of this phenotype [14]. Mutating the NES rescued H3K79 hypomethylation and retained CALM‐AF10 and DOT1L in the nucleus, suggesting that CALM‐AF10 results in mislocalization of DOT1L to the cytoplasm, thereby inducing global H3K79 hypomethylation [14].

Global H3K79 hypomethylation in *PICALM::MLLT10* leukemias promotes chromosomal instability, with hypomethylated cells displaying poor survival and frequent chromosome breaks and gaps after exposure to γ‐irradiation [40]. *PICALM::MLLT10* leukemias have been shown to be more prone to secondary chromosomal rearrangements than other leukemias with a fusion gene, such as *KMT2A::MLLT3* and *PML::RARA* leukemias [40]. Jones *et al*. [50] found a similar correlation between H3K79 hypomethylation and chromosomal instability, reporting that H3K79 hypomethylation was accompanied by aneuploidy and telomere elongation in Dot1L deficient mouse embryonic stem cells. The mechanism by which H3K79 hypomethylation induces chromosomal instability in *PICALM::MLLT10* leukemia remains unidentified, and the effect of DOT1L mislocalization on DNA repair processes requires further investigation [48].

### Target gene H3K79 hypermethylation

Paradoxical to its role in global hypomethylation, DOT1L induces local H3K79 *hyper*methylation at target loci in *PICALM::MLLT10* leukemias. While CALM‐AF10 mislocalizes DOT1L to the cytoplasm in CALM‐AF10‐expressing MEFs, DOT1L is not completely removed from the nucleus, with 44% of cell nuclei retaining both DOT1L and CALM‐AF10 [14]. Furthermore, CALM‐AF10 may re‐enter the nucleus via the nuclear localization signal conserved in its AF10 domain [1, 27, 31]. Accordingly, Okada *et al*. [33] have detected nuclear colocalization of CALM‐AF10 and DOT1L in CALM‐AF10‐expressing cells. This interaction facilitates local H3K79 hypermethylation at target loci, including leukemogenic *HOXA* cluster genes [33, 41]. Conway *et al*. [14] additionally detected H3K79 hypermethylation at *Hoxa7, Hoxa9, Hoxa10*, and *Hoxa11* promoters in a *PICALM::MLLT10* mouse leukemia model.

Consistent with findings that H3K79 methylation occurs at sites of active transcription, genes at these hypermethylated loci are upregulated. A mouse model of *PICALM::MLLT10* leukemia displayed overexpression of *Hoxa5, Hoxa7, Hoxa9*, and *Hoxa10* [30]. Similarly, patient T‐ALL samples showed overexpression of *Hoxa5*, *Hoxa9*, and *Hoxa10* [58]. In addition to these *HOXA* genes, patient T‐ALL samples exhibited overexpression of *MEIS1* and *BMI1*; the *PICALM::MLLT10* mouse model also overexpressed *Meis1* [30, 58]. *Meis1* cooperates with *Hoxa9* in myeloid and *KMT2A*‐rearranged leukemias [59, 60]. MEIS1 is essential for normal hematopoiesis and acts to prevent apoptosis, promote proliferation, and maintain the hematopoietic stem cell population [61]. While comparisons are often made between *KMT2A* rearranged leukemias and *PICALM::MLLT10* leukemia due to similarities in the transcriptional profile, there are distinctions between the two groups. For example, while both present with upregulated expression of *HOXA* and *MEIS*, *PICALM::MLLT10* translocated leukemias also demonstrate high *BMI1* expression [62]. BMI1 is part of Polycomb repressive complex 1 (PRC1), which is an E3 ubiquitin ligase that mono‐ubiquitinates H2A at lysine residues 118 and 119 [61, 62]. *BMI1* expression is important for repressing developmental genes and regulating the maintenance of stem cells, and it has been shown to be required by CALM‐AF10 to induce a leukemogenic transformation [24, 62, 63].

Further linking H3K79 hypermethylation to gene overexpression, Dot1L knockout reduced *Hoxa5, Hoxa7, Hoxa9, Hoxa10*, and *Meis1* expression in *PICALM::MLLT10* transformed murine bone marrow cells [64]. Pharmacologic inhibition of Dot1L led to similar effects in *Hoxa9* and *Meis1* expression [64]. Conway *et al*. [14] demonstrated that, as with global hypomethylation, the CALM‐AF10 NES is both necessary and sufficient for both *HOXA* overexpression and H3K79 hypermethylation. Expression of a mutated NES abolished these effects and fusion of only the NES to AF10 resulted in *HOXA* overexpression and H3K79 hypermethylation comparable to cells transformed with full‐length *PICALM::MLLT10* [14].

### The leukemogenic impact of epigenetic alterations

Importantly, DOT1L influences leukemogenic potential through these epigenetic modifications. Mouse bone marrow cells retrovirally transduced with *PICALM::MLLT10* lacking the Dot1L‐interacting OM‐LZ domain could not sustain colony formation in serial colony‐plating assays, while cells transduced with wild‐type *PICALM::MLLT10* continued to form colonies through the third round of plating [33]. Likewise, *PICALM::MLLT10* transformed bone marrow cells derived from a Dot1L knockout mouse model lacked H3K79 di‐methylation, promoting the formation of differentiated colonies in methylcellulose [64]. Similarly, the DOT1L inhibitor EPZ004777 decreased cell viability and colony‐forming potential *in vitro* and *in vivo* in *PICALM::MLLT10* transformed mouse bone marrow cells [64].

Despite the identification of *HOXA*, *MEIS*, and *BMI1* as critical oncogenes upregulated by CALM‐AF10 to promote leukemogenesis, a full understanding of cooperating mutations in this disease is lacking. More recent studies have included genomic profiling of *PICALM::MLLT10* in AML and T‐ALL leukemia samples and demonstrate a myriad of additional mutations which contribute to pathogenesis [65]. Future studies should additionally explore potential synergism of known epigenomic alterations in *PICALM::MLLT10* translocated leukemia, with the multiple dimensions of chromatin states on epigenetic regulation of gene expression in cells carrying the CALM‐AF10 translocation fusion protein.

## Endocytosis and the plasma membrane

Given the role of endogenous CALM in promoting CME, oncogenic CALM‐AF10 has the potential to disrupt cellular processes that are dependent on CME. While several CME events can be affected by CALM‐AF10, the most well‐described effect is on iron homeostasis, which serves as a model for how trafficking of other macromolecules at the plasma membrane may be impacted by CALM‐AF10 [66]. Transferrin (Tf) is a protein responsible for delivering iron from the blood to target cells, which is achieved when Tf binds to cells expressing transferrin receptors (TfRs) [67]. The Tf/TfR complex is endocytosed via CME, which ultimately releases iron intracellularly and recycles the TfR to the plasma membrane [67]. In studies examining the role of endogenous CALM in normal hematopoiesis and iron homeostasis, it was determined that CALM is required for proper blood cell development and intracellular iron delivery in mice [8, 66]. In studies using *picalm* ^−/−^ knockout mice, the loss of CALM induced anemia, growth retardation, splenomegaly, and dysregulated hematopoiesis [66]. This impact on normal hematopoiesis in *picalm* ^−/−^ mice was most pronounced in erythroid cells, which demonstrated a decreased ability to both internalize TfRs and develop into mature erythroblasts [66]. Impaired iron homeostasis was also observed in studies using murine embryonic fibroblasts (MEFs), wherein a knockout of *PICALM* resulted in reduced TfR internalization [8, 10, 66]. In *picalm* ^−/−^ MEFs, transferrin internalization was shown to be rescued by transduction with endogenous CALM, but not with oncogenic CALM‐AF10 [10]. The inability of CALM‐AF10 to rescue this phenotype suggests that CALM‐AF10 does not maintain endogenous CALM's ability to promote CME [10]. This hypothesis is further supported by studies showing that wild‐type MEFs (*picalm* ^+/+^) demonstrate increased levels of serum transferrin when transduced with CALM‐AF10, but not endogenous CALM [10]. These results additionally suggest that CALM‐AF10 exerts a dominant‐negative effect on endogenous CALM's ability to facilitate CME [10]. In summary, these studies highlight the importance of CALM in facilitating CME, and the impact of CALM‐AF10 in dysregulating cellular processes dependent on CME, such as iron homeostasis.

## Clinical considerations

t(10;11)(p12‐12;q14‐21) *PICALM::MLLT10* translocations have been identified in T‐ALL, AML, and, in rare cases, AUL, MPAL, and B‐ALL. A study reported that out of 144 patients with *PICALM::MLLT10* translocated leukemia, 65% were diagnosed with T‐ALL, 39% with AML, 8% with ALAL including MPAL and AUL, and 0.7% with B‐ALL [68]. The translocation is most commonly found in T‐ALL, being detected in ~6–7% of cases [65, 69]. Among T‐ALL cases, *PICALM::MLLT10* expression is largely restricted to immature TCR γ/δ and TCR γδ phenotypes [70]. The incidence of *PICALM::MLLT10* in AML is much lower, being detected in less than 1% of pediatric cases [65].

### High‐risk factors in *PICALM::MLLT10* Leukemia

Patients with *PICALM::MLLT10* translocated leukemia tend to present with additional high‐risk features. The presence of extramedullary disease, including central nervous system (CNS) disease, has been reported in several studies [65, 71, 72]. CNS involvement has been reported at a higher rate in *PICALM::MLLT10* leukemias and is an indicator of a poor prognosis [71]. One of the largest studies to date assessed 98 *PICALM::MLLT10+* patients with ALL, AML, AUL, or MPAL and reported that extramedullary disease was present in 12% of cases [71]. Another study detected extramedullary disease in 70% of *PICALM::MLLT10+* AML cases and 75% of *PICALM::MLLT10+* T‐ALL cases [65]. Finally, Sun *et al*. [73] reported the presence of extramedullary disease in 50% of *PICALM::MLLT10+* patients with ALAL, T‐ALL, AML, B‐ALL, and MPAL. In contrast, extramedullary disease is present in approximately 20% of an unselected population of ALL patients and 23.7% of unselected AML patients [74, 75].

*PICALM::MLLT10* leukemia patients frequently present with significantly elevated white blood cell counts (> 50 K), which is a known adverse prognostic factor in ALL. A study looking at 14 patients diagnosed with *PICALM::MLLT10* T‐ALL reported that 35.7% of cases had a white blood count (WBC) of over 100 000·μL^−1^ [69]. A high white blood cell count has also been frequently detected in *PICALM::MLLT10+* patients with AML, as one study reported 44.4% of cases having over 100 000·μL^−1^. That study also suggested high white blood cell counts among *PICALM::MLLT10+* T‐ALL cases; however, only 12.5% of cases exceeded 100 000·μL^−1^ [71].

The detection of additional cytogenetic aberrations is a third high‐risk feature in many *PICALM::MLLT10* leukemia patients. The rate of additional aberrations in *PICALM::MLLT10+* T‐ALL and AML was 20% and 51% respectively, according to one study. The study also reported additional abnormalities in patients with AUL and MPAL. RAS pathway mutations, *NOTCH1* mutation, *WT1* mutation, and *ETV6* deletion were the most common abnormalities detected [71]. Another study performed cytogenetic analysis on seven *PICALM::MLLT10+* AML cases and one AUL case and found additional aberrations in all eight patients [65]. A third study found additional mutations in five (one AML, three ALL, and one MPAL) out of six (two AML, three ALL, one MPAL) *PICALM::MLLT10+* leukemia cases [72]. Overall, the presence of these additional high‐risk features in *PICALM::MLLT10* leukemias contributes to their poor prognosis. A summary of published clinical outcomes in *PICALM::MLLT10* leukemia is presented in Table 1.

**Table 1:** Clinical outcomes in *PICALM::MLLT10* translocated leukemia. The survival of pediatric patients with *PICALM::MLLT10* translocated ALL treated with chemotherapy +/− HSCT exceeds the survival of pediatric patients with *PICALM::MLLT10* AML treated with chemotherapy +/− HSCT [71]. The survival of pediatric patients with *PICALM::MLLT10* AML is not improved by HSCT. The 5‐year overall survival of pediatric patients with *PICALM::MLLT10* translocated acute leukemia exceeds adult survival [68]. The addition of venetoclax to chemotherapy demonstrates promising response rates in *PICALM::MLLT10* translocated acute leukemias [73].

Population	*n*	Treatment	Outcome	References
Pediatric ALL	49	Chemotherapy	5‐year OS 77%	[71]
	6	Chemotherapy + HSCT	5‐year OS 60%	[71]
Pediatric AML	26	Chemotherapy	5‐year OS 36%	[71]
	13	Chemotherapy + HSCT	5‐year OS 37%	[71]
Pediatric AL	52	Chemotherapy +/− HSCT	5‐year OS 72%	[68]
Adult AL	92	Chemotherapy +/− HSCT	5‐year OS 33%	[68]
Adult T‐ALL	6	Chemotherapy	CR 50%	[73]
	4	Chemotherapy + Venetoclax	CR 100%	[73]
Adult AML	3	Chemotherapy	CR 33%	[73]
	1	Chemotherapy + Venetoclax	CR 100%	[73]
Adult ALAL	5	Chemotherapy	CR 0%	[73]
	6	Chemotherapy + Venetoclax	CR 83%	[73]
Adult B/T‐ALL	1	Chemotherapy	CR 0%	[73]
	1	Chemotherapy + Venetoclax	CR 0%	[73]
Adult B‐ALL	1	Chemotherapy	CR 100%	[73]

### Prognosis in *PICALM::MLLT10+* patients

**Fig. 3:**
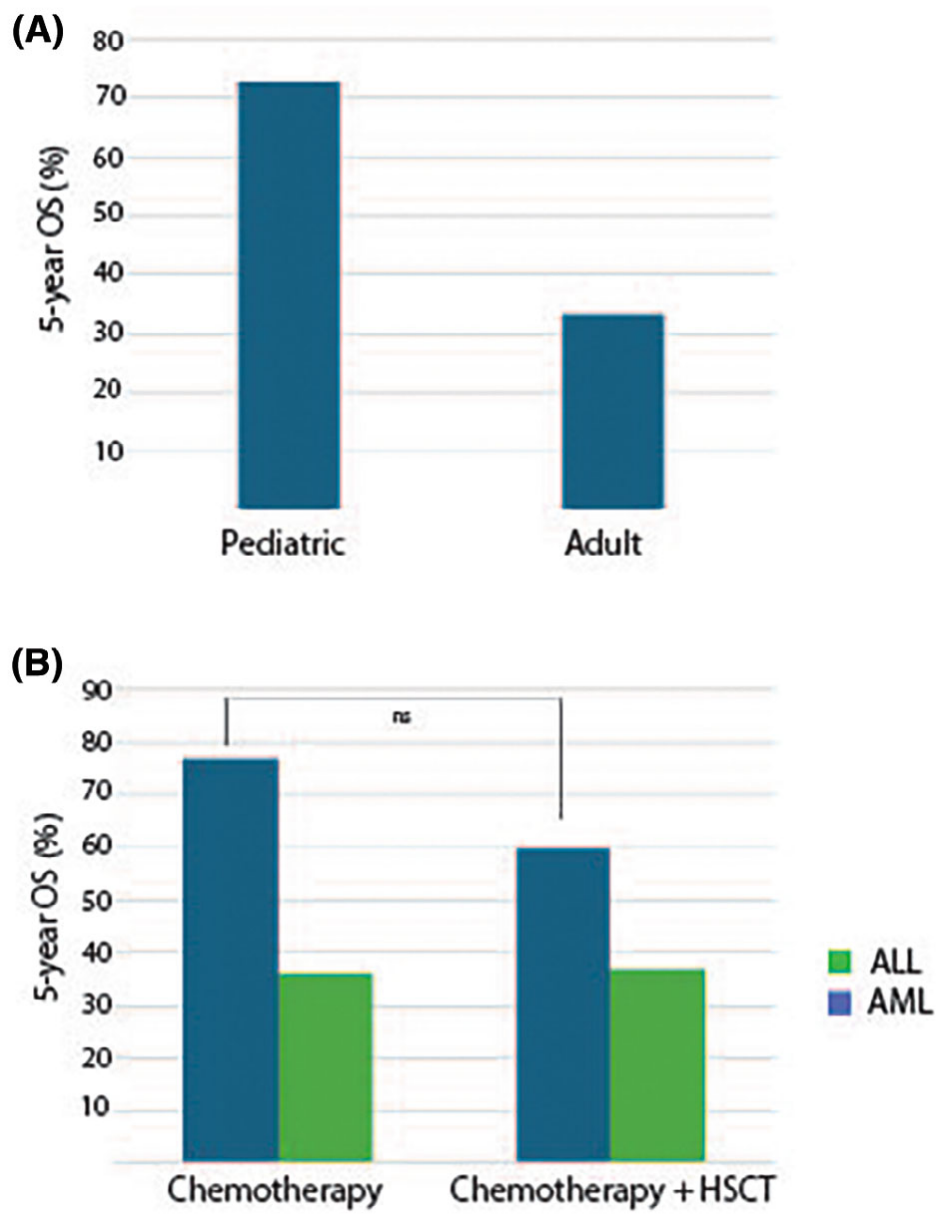
Clinical outcomes in *PICALM::MLLT10* translocated leukemia. (A) The 5‐year overall survival of pediatric patients with *PICALM::MLLT10* translocated acute leukemia exceeds adult survival [68]. (B) The survival of pediatric patients with *PICALM::MLLT10* translocated ALL treated with chemotherapy +/− HSCT exceeds the survival of pediatric patients with *PICALM::MLLT10* AML treated with chemotherapy +/− HSCT. However, the survival of pediatric patients with *PICALM::MLLT10* leukemia is not significantly improved by HSCT [71].

Among *PICALM::MLLT10*+ leukemia cases, adult patients have a worse prognosis than pediatric patients. A study assessing the survival of patients with *PICALM::MLLT10*+ leukemia, including T‐ALL, AML, MPAL, AUL, and B‐ALL, reported a 5‐year OS of 33% for adult patients and 73% for pediatric patients (Fig. 3A) [68]. These findings suggest that the role of the *PICALM::MLLT10* fusion in patient outcomes is age dependent.

The presence of the CALM‐AF10 fusion protein in AML patients results in a worse prognosis compared to an unselected AML patient population. A study assessing unselected AML outcomes in children reported a five‐year OS rate of 48.9% for patients enrolled on protocol AML97 and 71.2% for patients enrolled on AML02. The study also reported a five‐year EFS rate of 43.5% for patients enrolled on AML97 and 61.8% for patients enrolled on AML02 [76]. Several studies assessing outcomes of *PICALM::MLLT10+* AML patients reported a five‐year OS rate of 26% and a five‐year EFS rate of 22.5% [65, 71]. The five‐year OS rate and five‐year EFS rate of *PICALM::MLLT10+* cases are much lower compared to both cohorts of unselected AML cases. These data indicate that AML patients with the CALM‐AF10 fusion protein represent a high‐risk group requiring aggressive and perhaps novel treatments.

Because of the prevalence of *PICALM::MLLT10* in T‐ALL, there have been several studies looking at the impact of the fusion protein on various subgroups of T‐ALL according to stage of differentiation. Some studies report that the presence of the fusion protein does not seem to have an impact on final outcomes. For example, one study reported that there was no statistically significant difference between *PICALM::MLLT10+* and *PICALM::MLLT10‐* outcomes, with five‐year EFS of 71.4% and 62.5%, respectively [69]. However, several other studies have found that CALM‐AF10 does indeed impact T‐ALL outcomes [69]. These contradictory findings suggest a need for further research on the influence of the CALM‐AF10 fusion on T‐ALL outcomes. A clearer impact of *PICALM::MLLT10* on prognosis in T‐ALL seems to be dependent on lineage and stage of maturation. As discussed above, *PICALM::MLLT10* is primarily detected in T‐ALL with a TCR γ/δ phenotype or immature TCR δ/γ phenotype. Several studies have found that T‐ALL with an immature phenotype independently identifies a patient population with a poor prognosis [69, 70]. One study reported that 3/3 adults with *PICALM::MLLT10+* TCR γ/δ T‐ALL reached complete remission 46–64 months from diagnosis, whereas 8/9 adults with immature *PICALM::MLLT10+* T‐ALL died from their disease. Additionally, 5/5 children in the study with *PICALM::MLLT10+* TCR γ/δ T‐ALL remain in remission 8–115 months from induction, whereas 2/3 children with immature T‐ALL have relapsed. The concurring evidence across age groups and studies indicates that immature T‐ALL characterized by the *PICALM::MLLT10* translocation represents a high‐risk population.

### ETP T‐ALL: A subtype of *PICALM::MLLT10* Leukemia

Early T‐cell precursor acute lymphoblastic leukemia (ETP‐ALL) is a subtype of T‐ALL derived from thymic cells at the ETP differentiation stage. ETP‐ALL makes up approximately 15% of pediatric T‐ALL cases and 25% of adult T‐ALL cases. Cytogenetically unselected ETP‐ALL was originally found to have a worse prognosis than unselected non‐ETP‐ALL in both adult and pediatric cases [77]. More recently, several studies have concluded that there is no significant difference in OS between unselected ETP‐ALL and non‐ETP‐ALL in both pediatric and adult cases [68]. One study investigated the impact of the CALM‐AF10 fusion on ETP‐ALL vs non‐ETP‐ALL outcomes. The investigators found that adult but not pediatric *PICALM::MLLT10+* ETP‐ALL patients demonstrated inferior survival compared to *PICALM::MLLT10+* non‐ETP‐ALL patients. Adult *PICALM::MLLT10 +* ETP‐ALL patients had a survival probability of ~0.12 at 50 months compared to ~0.65 in *PICALM::MLLT10+* non‐ETP‐ALL patients at the same time point. Pediatric *PICALM::MLLT10+* ETP‐ALL patients have a survival probability of ~0.75 at 50 months compared to ~0.8 in *PICALM::MLLT10+* non‐ETP‐ALL patients at the same time point [68].

### Treatment strategies for *PICALM::MLLT10* Leukemia

Mark *et al*. [71] identified that hematopoietic stem cell transplantation (HSCT) did not increase survival in pediatric patients diagnosed with *PICALM::MLLT10* translocated AML or ALL (Fig. 3B). The study reported that *PICALM::MLLT10*+ ALL patients who received HSCT had a 5‐year projected OS of 60% compared with a 5‐year OS of 77% in those not treated with HSCT. Similarly, *PICALM::MLLT10*+ AML patients who received HSCT had a projected 5‐year OS of 37%, which was not improved compared with *PICALM::MLLT10*+ AML patients who did not receive HSCT and had a projected 5‐year OS of 36% [71].

The poor prognosis associated with *PICALM::MLLT10*+ acute leukemias suggests a need for novel treatment options. DOT1L inhibitors have been a promising targeted therapeutic option, given the role of DOT1L in AF10‐translocated leukemogenesis. A small molecule inhibitor of DOT1L, EP2004777, was used to treat *KMT2A::MLLT10* and *PICALM::MLLT10* transformed cells *in vitro* and yielded encouraging results. Treatment led to the suppression of leukemogenic genes such as *Hoxa* and *Meis1* and impaired proliferation of *KMT2a::MLLT10* and *PICALM::MLLT10* transformed cells [64]. However, DOT1L inhibitors have not yielded similar results in clinical trials. One study confirmed the inhibition of DOT1L in leukemic blasts by pinometostat but reported no evidence to confirm that it was an effective novel agent relative to standard care [78].

More recent studies have assessed the potential clinical benefits of the BCL2 inhibitor venetoclax. *PICALM::MLLT10* leukemias demonstrate increased expression of BCL2, indicating that a BCL2 inhibitor could be effective. Several *PICALM::MLLT10+* patients received venetoclax after a poor response to standard treatment and demonstrated positive responses. Initially, six T‐ALL, 1 B‐ALL, and 1 B/T‐ALL patients received standard T‐ALL induction chemotherapy, five patients with AML or ALAL received standard AML induction chemotherapy, two ALAL patients received standard AML induction therapy with VP regimen, and one ETP‐ALL patient received hyper‐CVAD. There were ETP‐ALL and ALAL patients (one of each) who initially received venetoclax along with other therapies that achieved complete remission. Of the 16 patients who did not initially receive venetoclax, only five achieved complete remission. Of the 11 patients who were not in remission at the end of standard induction therapy, five received combination therapy with venetoclax and achieved complete remission [73]. Due to the positive impact venetoclax had on patient outcomes, the study concluded that this targeted therapy could prove to be an effective treatment when combined with chemotherapy followed by HSCT for patients with *PICALM::MLLT10+* acute leukemia [73].

## Conclusions

*PICALM::MLLT10* translocations are a poor prognostic factor in acute leukemia, with suboptimal survival rates, even with intensive chemotherapy. The lack of response to HSCT demonstrates that novel therapeutic approaches to the leukemia subtype are desperately needed. Over the past 30 years, we have gained a deeper understanding of the molecular mechanisms underlying *PICALM::MLLT10* translocated leukemias. Global H3K79 hypomethylation is complemented by DOT1L mediated focal H3K79 hypermethylation at specific chromatin loci, including the *HOXA* gene cluster, which determines a transcriptional program driving the pathogenesis of disease. Components of the nuclear pore complex may influence subnuclear chromatin localization and transcription of genes critical for *PICALM::MLLT10* mediated leukemogenesis. Evaluation of mechanisms of epigenetic control and transcriptional dysregulation has already led to clinically relevant discoveries and early phase clinical trials. For example, DOT1L inhibitors demonstrated promising preclinical results; though unfortunately, they did not demonstrate significant benefit in clinical trials. More recently, the BCL2 inhibitor venetoclax has shown promising results in small case series of patients with *PICALM::MLLT10* leukemia and is poised to be tested as a rational molecularly targeted therapy in larger prospective trials.

Furthermore, altered subcellular localization of critical proteins including nucleocytoplasmic shuttling of the fusion oncoprotein itself and perturbations of the plasma membrane proteome are increasingly recognized as contributors to the biology of this leukemia. The interaction of leukemia cells with the tumor microenvironment is increasingly recognized to play a role in chemotherapy resistance, development of CNS disease, and leukemic relapse. Altered leukemic plasma membrane dynamics may contribute to these poor outcomes. Future directions in clinically relevant research in *PICALM::MLLT10* leukemia include exploration of targeting the altered CALM‐AF10 plasma membrane proteome with therapeutic intent.

The enhanced appreciation of the complex biological mechanisms underlying poor outcomes in patients with *PICALM::MLLT10* leukemias provides ample opportunity for the development of additional rationally targeted therapeutics and points toward the next steps forward in improving outcomes in this high‐risk population.

## Author contributions

JMC, ACN, NB, HH, and JLH wrote the article. JMC, HH, and JLH created the figures and table. GSS, JLS, and DSW critically reviewed and revised the article.
